# A qualitative study of maternal and paternal parenting knowledge and practices in rural Mozambique

**DOI:** 10.1186/s12889-024-19291-2

**Published:** 2024-07-03

**Authors:** Marilyn N. Ahun, Lilia Bliznashka, Svetlana Karuskina-Drivdale, Gino Regina, Aisha K. Yousafzai, Joshua Jeong

**Affiliations:** 1 Department of Medicine, Faculty of Medicine and Health Sciences, McGill University, Montréal, H4A 2S5 5252 boulevard de Maisonneuve, 2nd Floor, Canada; 2 Department of Global Health and Population, Harvard T.H. Chan School of Public Health, Boston, USA MA; 3 Department of Social and Preventive Medicine, Université de Montréal School of Public Health, Montréal, Canada QC; 4 International Food Policy Research Institute, Washington D.C., USA; 5 PATH, Maputo, Mozambique; 6 Hubert Department of Global Health, Rollins School of Public Health, Emory University, Atlanta, USA

**Keywords:** Nurturing care, Families, Parenting, Early child development, Mozambique

## Abstract

**Background:**

Providing nurturing care for young children is essential for promoting early child development (ECD). However, there is limited knowledge about how mothers and fathers across diverse contexts in sub-Saharan Africa care for their children and from whom they receive guidance and support in their caregiving roles. We aimed to examine caregivers’ nurturing care practices and sources of parenting knowledge in rural Mozambique.

**Methods:**

This is a secondary analysis using data from a qualitative evaluation of a pilot intervention to improve nurturing care for early child health and development within existing health systems. The evaluation was conducted across three primary care health facilities and their catchment areas in Nampula province, Mozambique. For this study, we analyzed data from in-depth interviews conducted with 36 caregivers (32 mothers and 4 fathers) to investigate mothers’ and fathers’ daily caregiving experiences. Data were analyzed using thematic content analysis.

**Results:**

Caregivers described various caregiving roles relating to general caregiving of young children (e.g., feeding, bathing, caring for child’s health) and stimulation (e.g., play and communication) activities. Mothers more commonly engaged in general caregiving activities than fathers, whereas both mothers and fathers engaged in stimulation activities. Other family members, including siblings, grandparents, and aunts/uncles, were also actively engaged in general caregiving activities. With respect to sources of parenting knowledge, caregivers received parenting guidance and support primarily from their own mothers/parents and facility-based health providers.

**Conclusions:**

These findings highlight the importance of adopting a holistic approach involving caregivers and their context and reveal potential strategies to promote caregiving and ECD in rural Mozambique and similar contexts.

**Supplementary Information:**

The online version contains supplementary material available at 10.1186/s12889-024-19291-2.

## Background

Nurturing care comprises a set of inter-related knowledge, skills, and behaviours that enable optimal caregiving for healthy early child development (ECD). These competencies are encapsulated in the Nurturing Care Framework, which highlights five components (good health, adequate nutrition, responsive caregiving, security and safety, and opportunities for early learning) through which public services can promote ECD. [1] Recently, there have been increasing efforts to implement public health interventions that target stimulating and responsive caregiving to improve ECD outcomes in low- and middle-income countries, with only a handful of studies conducted in sub-Saharan Africa. [2–4] Stimulation (i.e., engaging in play and communication activities) and responsive caregiving (i.e., being aware of a child’s signals and providing timely- and developmentally-appropriate responses) are positively associated with ECD. [2, 3, 5, 6] However, there is limited understanding of how caregivers across diverse contexts in sub-Saharan Africa engage with young children to promote their development.

Evidence to date on nurturing care across various contexts in sub-Saharan Africa largely comes from indicators collected in large-scale household surveys such as the Demographic Health Surveys (DHS) and Multiple Indicator Cluster Surveys (MICS). This study focuses on the caregiving context in Mozambique, where population-based data (DHS 2022–23 and DHS 2011) show that 32% of Mozambican households are headed by women. [7, 8] With respect to caregiving practices, 31% of adult household members engaged in stimulation activities with young children. [9] This was likely mothers and other caregivers in most cases, as only 16% of fathers reported engaging in any stimulating activity with their child – despite 72% of children living in a household with their biological father. However, some of these estimates are over a decade old, and no recent national data on parenting practices in the general population in Mozambique exists.

These data are comparable to data (2010–2018) indicating that 14.6% of mothers and 3.9% of fathers in the sub-Saharan African region engage in adequate stimulation activities with children under age 5 years. [10, 11] Comparatively, 36–70% of mothers and 12–21% of fathers in other regions of the world engage in stimulation activities with their children. A greater proportion of mothers engage in stimulation activities compared to fathers, likely reflecting the role of mothers as primary caregivers of young children in many countries across SSA and globally. [10, 12] Data from DHS and MICS also reveal that other family members (e.g., siblings, grandparents, aunts/uncles) play an important role in caring for young children. Across sub-Saharan Africa, approximately one in five (19.2%) other caregivers (i.e., household members older than 15 years) provide adequate stimulation to young children and are more likely to engage in activities such as playing or going out with the child compared to mothers or fathers. [11] There are no disaggregated data on other caregivers in the Mozambican MICS.

Overall, much of the evidence on caregiving practices in Mozambique – and across sub-Saharan Africa – is based on quantitative data. Although this information is helpful, it is limited by the lack of nuanced information on diverse caregiving and household arrangements across and within countries, including how mothers and fathers interact with their children daily and from whom they receive guidance and support in caring for children. Only two reports have provided such nuanced data in the Mozambique context. [13, 14] Using qualitative methodologies, the reports highlight the primary role of mothers in feeding, bathing, and caring for the child’s health, and describe fathers’ responsibilities as buying food, clothes, and medicine. Both mothers and fathers were reported to engage in stimulation activities with their child, with most engaging in communication activities. (e.g., talking, singing, telling stories).

These qualitative approaches capture the lived experiences of caregivers of young children and can help elucidate how caregivers ascribe meaning to their experiences. However, these – and the handful of other qualitative reports from diverse settings across sub-Saharan Africa [15–17] – focused solely on mothers, thus limiting our understanding of how fathers perceive theirs and their partner’s caregiving roles. Further qualitative research is needed to examine the perspectives of fathers and the caregiving roles of other family members and to understand the sources of guidance and support mothers and fathers receive as caregivers of young children. Understanding the nurturing care practices and sources of parenting knowledge from the perspectives of mothers and fathers is important considering how parenting is influenced by culture and gender norms within a given context. [18] Such information can highlight intervention leverage points and inform the implementation of locally-acceptable strategies to improve nurturing care practices and ECD. [19]

This study aims to address existing gaps in the parenting literature on Mozambique and across sub-Saharan Africa more generally by qualitatively investigating caregivers’ nurturing care practices and the sources of their parenting knowledge in rural Mozambique. We used a phenomenological design, thus enabling us to explore the essence of nurturing care by studying the lived experiences, perceptions, and personal meanings of mothers and fathers in this context. [20]

## Methods

### Study design and setting

This study was conducted in Monapo district, Nampula province as part of a larger qualitative study that evaluated the delivery, acceptability, and perceived impacts of a pilot intervention to improve nurturing care for early child health and development within existing health systems (see Supplementary Material for a summary). [21] Prior publications from this study have examined barriers and facilitators to father involvement in early child health services [12] and the effects of the COVID-19 pandemic on child health service delivery and use. [22] The present sub-study leveraged data collected on mothers’ and fathers’ caregiving experiences during the pilot intervention to help understand caregivers’ nurturing care practices and sources of parenting knowledge in rural Mozambique. This sub-study therefore consists of a secondary analysis of the qualitative data collected in the larger study. The COREQ checklist guided reporting of the methodology and study results. [23]

Nampula – located in northeastern Mozambique – has some of the poorest nutrition and health indicators for children under 5 years of age in the country, with high rates of child malnutrition (stunting [55%], wasting [6.5%], and anemia [73%]). [24] Education levels are lower than the rest of the country with 4.9% of women and 14.9% of men completing primary education. [7] The primary source of income for most households is farming and agricultural activities. [7] The prevalence of caregiving practices in Nampula is comparable to national prevalence rates: 34% of household members engage in adequate levels of stimulation and 19% of fathers engage in any stimulating activity with young children. [9]

Most caregivers in Nampula have access to child health services from local health facilities and community health workers. In health facilities, a variety of providers – including maternal and child health nurses and nutritionists (henceforth referred to as facility-based providers) – provide consultations in children’s health. At the community level, health services are provided by community health workers (henceforth referred to as community-based providers) trained in general healthcare and local non-governmental organizations that focus on water, sanitation, and hygiene, and supporting children with malnutrition. Over the past decade, the Ministry of Health has made substantial investments in Nampula’s health system, particularly with regard to integrating child nutrition services into primary healthcare services. [25] Since 2014, there have also been increasing efforts to integrate nurturing care interventions – with a focus on promoting ECD – into existing facility- and community-based health services at national and provincial levels. [21]

### Data collection

Per the main study design, caregivers were sampled from three health facilities and their catchment areas in Monapo district, Nampula province. First, 6–7 caregivers were randomly selected for household interviews from a list of all caregivers who had attended a well-child or sick-child visit in the month prior to data collection. Second, an additional 5–6 caregivers per health facility were recruited during random visits to each of the facilities and interviewed as they exited. Interviews lasted, on average, about one hour. Eligibility criteria for caregivers were adult (≥ 18 years) primary caregiver of a child < 2½ years who resided in the same household as the child, caregiver’s household was located within the geographic catchment area of the health facility, caregiver visited the health facility for child health service in the past month, and caregiver provided informed consent for study participation. Caregivers were interviewed by a team from Maraxis B.V. – a local research firm with prior experience conducting surveys and qualitative research on maternal and child health – which consisted of three Mozambican research assistants (one male, two females) with bachelors’ degrees Two of the interviewers were from Nampula province, where the study was conducted, and were fluent in Makua and Portuguese, and the other two were from Maputo province and fluent in English and Portuguese. The field team fostered an inclusive environment for learning from respondents rather than merely obtaining information or portraying the perception that a respondent was being evaluated. This study only uses data from interviews with caregivers.

Data were collected between October and November 2020 using a semi-structured topic guide developed for the larger study that included questions focused on understanding caregivers’ engagement in the pilot intervention. The guide also included questions about respondents’ caregiving experiences, including their daily activities and routines with the child, the perceived caregiving roles of mothers, fathers, and others in the household/extended family (e.g., “*Describe your role as your child’s caregiver?” “What are your main responsibilities as a caregiver in a typical day?” “What is your child’s father/mother’s role?*”) and what guidance and support caregivers received with respect to how to care for their child (e.g., “*How did you learn how to take care of a child?” “Did you receive information on how to take care of your child?*”).

Research assistants received a five-day (virtual) training on qualitative research methods, ethical conduct of research, and use of the topic guide led by the senior author (JJ). The topic guide was pilot tested for three days prior to data collection and refined as needed. Interviews were conducted face-to-face in respondents’ preferred language (Portuguese or Makua) in a private location at a time when health facility consultations were returning to pre-pandemic levels. [22] Nevertheless, research assistants adhered to strict COVID-19 risk mitigation protocols (i.e., physical distancing, outdoor setting, provision and use of face masks, and water and soap for handwashing). All interviews were audio-recorded, transcribed, and translated verbatim into English by the research assistants. Transcripts were independently reviewed by the field research manager daily to ensure completeness and accuracy, and to make revisions as needed. JJ debriefed daily with the field research team via Zoom to discuss data collection progress, emerging findings, and assess whether data were reaching saturation (i.e., point at which no new information was obtained). Further details on the methodology have been published elsewhere. [21]

### Data analysis

The initial codebook was developed by (JJ) based on the interview guide. This codebook was piloted and iteratively refined – using an inductive process – by three analysts (MNA, LB, JJ) who independently coded, annotated, and analyzed each English transcript line-by-line using NVivo (Version 12). [26] For this sub-study, we focused on two parent codes: parenting practices and sources of parenting knowledge. The analysts inductively identified topics within these parent codes and other relevant data shared by caregivers. Data analysis focused on meaningful units that richly describe the topic of interest using thematic content analysis. [27] Weekly meetings were held throughout the analysis process (November 2020-January 2021) to discuss codes, resolve any disagreements, review memos, and discuss emerging themes – informed by codes and the ideas generated within them – through a consensus building process. Discussions were also held with co-authors living in Mozambique (SD and GR) to support data interpretation. Through this process, we reviewed supporting evidence for each topic and between multiple topics to contextualize themes and generate findings.

### Research team and reflexivity

Throughout fieldwork, the team was in daily communication with JJ (a male Korean-American academic researcher with prior qualitative field experience in Eastern Africa), which enabled real-time discussions and reflexivity on the data. This process also enabled the team to provide inputs on the local cultural context to enhance the trustworthiness of interpretation and representation of findings.

JJ worked with MNA (a female Ghanaian academic researcher) and LB (a female Bulgarian academic researcher) – all three based at the same US academic institution at the time of data analysis – to analyze the data. MNA and LB also have prior field experience with qualitative studies in Eastern and Western Africa and all three data analysts have substantive PhD-level expertise in global public health, with a focus on caregiver engagement for the promotion of ECD. None of the data analysts travelled to Mozambique due to the COVID-19 pandemic. A subsample of transcripts was shared with co-authors (a female British-Pakistani, US-based global health professor with extensive qualitative research experience in Eastern Africa and studying parenting programs [AKY], a female Ukrainian PhD-level regional senior ECD specialist at a non-governmental organization in Mozambique [SD], and a male Mozambican provincial ECD coordinator with implementation experience in Mozambique [GR]) to discuss initial themes and help finalize the codebook. Overall, the diverse experiences and perspectives, along with the collaborative approach, yielded a thorough and balanced interpretation of the data.

### Ethical considerations

The research protocol was approved by the Institutional Review Boards of Harvard T.H. Chan School of Public Health and the Bioethics Committee of the Mozambican Ministry of Health. Informed consent forms were read aloud in Portuguese or Makua and participants either provided their signatures or thumbprints to indicate consent.

## Results

Thirty-six caregivers were interviewed: 32 mothers, 3 fathers, and 1 uncle who self-identified as the child’s father. Henceforth, we refer to these four male caregivers as fathers. Twenty interviews were conducted in participants’ homes and 16 at health facilities. On average, caregivers were 24 years old (standard deviation [SD] = 4.2, range = 18 to 36 years) and had 3.4 (SD = 1.8) children. The highest level of education for most caregivers (42%) was completed primary school. A quarter of the sample had completed secondary school, and the remaining 33% either completed some primary school (25%) or received no formal education (8%). Most caregivers (83%) stayed at home or worked in farms, with an average monthly household income of 2,482 Mozambican Meticals (~ 32.27 USD). Approximately half (53%) of target children were males, and the average age was 10.7 months (SD = 8.3).

Caregivers described how they cared for their child on a daily basis and from whom and how they received guidance and support about caregiving. For the parenting practices code, the topics discussed by caregivers included general caregiving activities (i.e., feeding, washing/bathing, caring for child when sick, ensuring child’s safety, disciplinary practices), stimulation activities (i.e., play and communication), and the specific parenting practices the different caregivers in their child’s life engaged in. This latter topic comprised the parenting practices of other caregivers (e.g., child’s grandparents, aunts/uncles, siblings) and the extent to which mothers or fathers engaged in certain parenting practices. For the sources of parenting knowledge code, caregivers described four primary sources of guidance and support: family, friends/neighbours, health providers (facility-based and community-based), and local media (i.e., means of mass communication such as TV, radio, etc.).

### Parenting practices

Caregivers described a variety of activities that they commonly engaged in to support the wellbeing of young children. We first present the general caregiving and stimulation activities mothers and fathers engaged in, then describe the parenting practices of other caregivers, and conclude by highlighting differences in the parenting practices mothers and fathers engaged in.

#### General caregiving activities

Caregivers consistently mentioned the following activities: bathing, washing and changing the child’s clothes, breastfeeding, preparing food for and feeding the child, taking the child to the health facility for routine care and when the child was ill, and maintaining a clean and safe home environment for the child.

“Interviewer: Describe to me your role as your daughter’s caregiver?

Respondent: When we wake up, I give her a bath, wash the clothes, feed her and when she is not feeling well, I take her to the hospital.

Interviewer: What are your main responsibilities as a care giver in a typical day?

Respondent: The first thing I do when she wakes up, I give her a bath, I prepare porridge for her and when she is not feeling well I take her to the hospital.

Interviewer: How do you take care of your daughter’s health?

Respondent: I take good care of her, I wash her clothes, give her a bath, feed her well so that she does not become sick and if it is in the morning, I prepare her some porridge mixed with grounded peanuts, I add some sugar and when I have milk I also add some.”

Caregivers also highlighted how they financially cared for their child’s by purchasing food, clothes, diapers, and other needs (e.g., medicine, bed nets). Even though we did not probe about disciplinary practices, when asked about how they care for their young children, two mothers noted that they did not use physical or verbal aggression to respond to their child when the child refused to feed or played with a dangerous object.

*“When my daughter errs or when she is playing with something that she should not, I forbid her to continue and when it is something that is safe, I leave her to play. That way, she can understand what she can do and what she cannot do. I do not beat my daughter or scream at her.”*

Mother-02, 19-to-24-month-old daughter

#### Stimulation activities

Beyond general caregiving activities, caregivers with older children (> 12 months) also described maternal and paternal engagement in stimulation activities, including play and communication. Although many caregivers described using household objects to make play materials for their child, few elaborated on whether and how they used these objects to interact with their child. In other words, it was not clear whether caregivers used these toys to engage in play with their children or whether the child played with the toy on their own. Some exceptions to this were one mother who described using a water bottle as a pretend phone with her child and one father who described kicking a ball with his child.

Caregivers also highlighted a variety of ways through which they communicated with their children. For example, caregivers described teaching children the names of family members, foods, and household objects. Several caregivers described more interactive styles of communication, such as giving instructions and encouraging the child to help them understand those concepts. A few caregivers also mentioned other activities such as singing, reading from books, and reciting poems.

“Interviewer: You told me you teach her how to play, where to step and not to step, where not to touch and to touch. What and how do you teach her?

Respondent: What I teach her while she is playing, I teach her to count, to complete words, she has a heavy tongue, so when she says, Pa.. I respond “papa” and from there she repeats the whole word, I teach her words and syllables in order not to stress her so that she will not withdraw from me.

Interviewer: What do you teach her to be an intelligent girl?

Respondent: I teach her many things. I teach her how to play with her brothers, she should not fight with her brother and when one of her brothers is angry I do tell him to apologize to her and say “sorry” this way I also teach her to say the same word to her brothers.”

Father-01, 13-to-18-month-old daughter

#### Parenting practices of other caregivers

While describing their caregiving roles, caregivers spontaneously mentioned other family members who supported the care of their children: older siblings, grandparents, and aunts/uncles. Older siblings were mentioned primarily in the context of playing with or supervising the child so that mothers and fathers could take care of other household tasks. Children’s grandmothers and aunts also engaged in general caregiving responsibilities, such as feeding the child, changing diapers, and accompanying mothers to child health visits. A few caregivers also mentioned that children’s uncles engaged in stimulation activities. One teenage single mother – who shared that the child’s father had abandoned them – highlighted how her father played a key role as a financial provider for her and her child’s needs, frequently played with the child, and accompanied her to health facility visits. In addition, adult family members were also described as sources of parenting support, from whom mothers and fathers received financial assistance, food, emotional support, and advice that in turn facilitated better care for children.

“Respondent: My daughter has not started eating. I only breastfeed her. I leave her with her brothers while I am doing other chores in the house, because these older ones eat and I also need to take a bath and change clothes.…

Interviewer: What do you do to prevent [name removed in transcript] becoming sick?

Respondent: I always ensure that I take care of her in the best way possible, giving her a bath so that she is not dirty, I always tell the brothers to play with her and not to let her play on the ground and I oblige them to sweep the compound and to always wash their hands while with her on the mat.”

Mother-03, 0-to-6-month-old daughter

“Interviewer: In terms of caring for her in terms of hygiene, what do you do?

Respondent: In terms of her hygiene, we all take care of that, her mother, I and the grandmother, gives her a bath three times a day, nowadays, she even requests for a bath and per day at times we give her a bath 5 times. When she takes a bath, she uses fresh clothes.

Interviewer: Who changes her diapers?

Respondent: I do and when I am not around her mother or her grandmother also do that.”

Father-01, 13-to-18-month-old daughter

*“Interviewer: What kind of food does she usually eat?*

*Respondent: I feed what we get. Her grandmother usually brings sweet potato, sometimes I make cornmeal porridge with moringa or peanuts that her grandmother cultivates and leaves here when she goes to the field. But she already eats thick porridge from cassava flour.*

*Interviewer: And what do you do when she is sick?*

*Respondent: I always take this child to the hospital. Sometimes her grandmother does…”*

Father-02, 19-to-24-month-old daughter

*“My father [child crying…] always gives me advice that in this world no one is an island and we need each other. In that regard, I should be respectful to all people. He always asks me whether I have gone to the health facility for my post-natal appointments and what I was told at the health facility. When I have an appointment at the health facility and I do not have money for transport, my father and my mother always help me. So that I should not miss and I can arrive at the hospital early.”*

Mother-04, 0-to-6-month-old son

### Differences in mothers’ and fathers’ parenting practices

Both mothers and fathers described general caregiving activities as primarily mothers’ responsibilities, whereas fathers’ caregiving contributions were primarily described as financial (e.g., purchasing foods, clothes, medicine). This division was also reflected in the roles of other caregivers, whereby grandmothers and aunts more commonly fed or bathed the child while grandfathers and uncles more commonly made financial contributions. When probed about the reasons behind these divisions in parenting practices, mothers and fathers explained that “women should take care of the children and men should go to work”, suggesting that caring for the child was broadly perceived as a maternal responsibility, whereas financial contributions were perceived as a paternal responsibility. However, in instances where fathers were absent from the child’s life (about one-third of the sample), mothers were the ones responsible for financial contributions.

“Interviewer: Why are there so many women [at the health facility] compared to men?

Respondent: Generally, in the pediatric consultation there are many women, because, I do not know if I will be very happy by describing, but the woman has been the person who takes good care of our children. So, they are the person who takes good care of our children. So, it is they who most often take our children to the hospital and on the other hand it can be because the men are working, so they have not had the opportunity to take their children to the hospital.”

Father-03, 19-to-24-month-old son

“Interviewer: Why are there few fathers accompanying their children to the hospital?

Respondent: The men do not have a lot of time, they have to go look for food for the children and when the baby is still very young, like my daughter, it has to be the mother taking the baby…Because [fathers] have to go and look for food for the children. They have a lot of things to deal with while the women come to the hospital, they go to the field. If they bring the child to the hospital and the child cries, can they breastfeed?”.

Mother-05, 0-to-6-month-old daughter

Despite these cultural norms, some fathers actively engaged in general caregiving activities, mostly in taking their child to the health facility. The active engagement of both mothers and fathers was also evident in descriptions of stimulation activities, where many caregivers shared that mothers and fathers engaged in play and communication activities with the child. It is worth noting that fathers were more commonly described as engaging in physically interactive stimulation activities (e.g., kicking ball, playing hide-and-seek) than mothers.

“Interviewer: Can you tell me about your role as a caregiver of your young child?

Respondent: When it dawns, I wash the face of my son and evaluate how he is by checking how he is acting when he wakes up, I bath him, change his nappy and ask the mother to breastfeed him, I cook for him porridge and I leave him to play with his brothers and sisters till he is tired or starts crying then I carry him on my back and when he is not feeling well I take him to the hospital.”

Father-04, 19-to-24-month-old son

“Interviewer: What does [name removed in transcript] father do to help with her growth and development?

Respondent: The father gives her a lot of attention. Most of the times when she comes back from the market, he brings her something like toys for her, a fruit, juice, cake, sugar for her porridge or for tea. I have also told her of what I heard from the hospital that we should talk to her normally so that she can learn the words. He does that with his daughter and he likes playing with her when he is back from his business. Interviewer: How does he play with her?

Respondent: He normally carries her on his back and runs. [name removed in transcript] likes playing the horse game with her father.”

Mother-02, 19-to-24-month-old daughter

Overall, both mothers and fathers expressed a desire for fathers to be more engaged in general caregiving activities because “the child has been created by two bloods” and thus it was the responsibility of both caregivers to care for the child.

“Interviewer: Why are you saying that it is a bad habit that men do not accompany their children to the hospital?

Respondent: I say that because some men it is because of their work and I do not blame that as much but there are situations should feel the responsibility of taking his own children to the hospital, especially when we know that the hospital has complicated attendance, we should stop for a bit our work so that we can understand about the health of our children. It is not all the time that the mothers can be able to explain all that they have been told at the hospital and we need to know and acknowledge that the mothers undertake a lot of household chores and we need to help”.

Father-01, 13-to-18-month-old daughter

“Interviewer: What could be done in order to increase the number of fathers to the health facility?

Respondent: They talk about porridge from sweet potatoes, porridge with peanuts, porridge with coconut, playing with the child, giving toys, showing them affection, talking to the babies, not beating them and not shouting at them.”

Mother-01, 0-to-6-month-old daughter

### Sources of parenting knowledge

The most frequently mentioned source of parenting knowledge was caregivers’ mothers, followed by facility-based health providers. Overall, it appeared that caregivers with lower levels of education (i.e., some or completed primary education) learned about caregiving almost exclusively from their mothers. Caregivers with higher levels of education (i.e., some or completed secondary education) mentioned multiple sources of knowledge, most frequently their mothers/parents and facility-based providers. More educated caregivers also provided more details on what type of messages they received from different sources, whereas less educated caregivers did not elaborate on the sources of specific types of parenting knowledge. For example, among caregivers who received guidance and support from multiple sources, their mothers, family, and friends were primarily a source of knowledge for general caregiving activities while facility-based providers provided guidance and support for stimulation activities.

“I learned [to raise a child] from my mother. […] she used to tell me that I have to give bath 3 times a day, I need to wash her clothes so that she is not dirty and ensure that she eats and always breastfeeding. [I have received information about parenting and how to take care of your child] Only from my mother.”

Mother-07, 0-to-6-month-old daughter

“In addition to my mother, I also learned [to care for a child] at the health center, every time I go, nurses always talk a little about everything, especially in terms of food, development, and the child’s wellbeing”

Mother-08, 19-to-24-month-old son

Community-based health providers and local media were mentioned as a source of information on nutrition. Some caregivers also recalled learning about the importance of playing and talking to their child from local radio campaigns and posters in the health facility. With respect to when knowledge was obtained, caregivers who identified their mothers/parents as a source shared memories from when they were a child themselves and had cared for a younger sibling. Caregivers who reported receiving guidance and support from facility-based providers shared that this occurred during both antenatal and postnatal consultations.

“I learned [how to care for a child] from the hospital when I was pregnant for the prenatal care, they always talked that we needed to speak to her while she was in the womb. When I gave birth to her, they were talking that we should play, talking to her and touching things so that she can see or hear. Show her things so that she could follow with her eyes so that we can know whether she can see. We should buy toys, give bath 3 times per day and always sleep under the mosquito net to avoid mosquito bites, breastfeed for 6 months and give cereal porridge with peanuts or coconut and not to use a lot of sugar. Avoid getting pregnant when the baby is still young.”

Mother-02, 19-to-24-month-old daughter

“As a father and a man, I learned to take care of my daughter through the experience that I had… I did not have the privilege to have all this care but I watch on the TV, what I see in the community, I feel that a child needs help especially that comes from home…I also copy or learn from her what her mother does and then I implement.”

Father-01, 13-to-18-month-old daughter

“Interviewer: What have you been hearing from your radio about early childhood care?

Respondent: They talk about porridge from sweet potatoes, porridge with peanuts, porridge with coconut, playing with the child, giving toys, showing them affection, talking to the babies, not beating them and not shouting at them.”

Mother-02, 19-to-24-month-old daughter

## Discussion

This qualitative study used data collected in the context of a pilot implementation evaluation to examine how mothers and fathers interacted with their children and from whom they received guidance and support for the care of their young children in rural Mozambique. Caregivers engaged in general caregiving activities and financially providing for their children, with mothers mostly engaging in the former and fathers in the latter. Other family members also engaged in these general caregiving activities. Both mothers and fathers engaged in stimulation activities such as making homemade toys for their child and talking and singing to them. With respect to sources of parenting knowledge, caregivers – especially those with higher levels of education – reported multiple sources, including their mothers and facility-based health providers. Overall, these findings provide important insights into parenting practices and sources of parenting knowledge in a low-income country context – rural Mozambique – that can help inform the development of locally-acceptable strategies to improve stimulating and responsive caregiving for ECD [4].

These findings align with prior qualitative research on nurturing care in rural areas of Eastern and Southern Africa. Despite the geographic, social, and cultural diversity of these settings, male and female caregivers (i.e., mothers, fathers, grandparents) in rural areas of Malawi, Tanzania, and other provinces in Mozambique, also identified mothers as the primary caregiver, especially in the first years of life [13, 14, 16, 28]. Nevertheless, as in our study, some caregivers expressed a desire for fathers to be more engaged in general caregiving activities and it was clear that some fathers were already engaged in stimulation activities [13, 14, 16, 28]. Another similarity across ours and previous studies is that when describing the stimulation activities they engaged in, caregivers more frequently mentioned talking with and singing to their child than playing with them. In our context, although caregivers provided toys for their children to play with, they did not report engaging in play themselves. While all types of play – including play that children engage in on their own – are valuable for ECD, children benefit from playing with adult caregivers as they provide more opportunities for complex and stimulating play. A potentially useful strategy for promoting more stimulating care, therefore, may be to encourage caregivers to play with their children and provide demonstrations and specific examples of how to do so [2]. That our findings are consistent with prior qualitative studies on parenting practices across diverse settings in Eastern and Southern Africa suggest that they could inform interventions in other contexts beyond rural Mozambique, and that findings from other similar contexts could be relevant for the rural Mozambican context.^15–17,28^

Paternal engagement in caregiving was acceptable to both mothers and fathers in our sample, and some fathers were willing to engage in general caregiving activities. This aligns with evidence from father-inclusive nurturing care interventions in East Africa [7, 28]. In a qualitative study from rural Rwanda, fathers discussed the impact of a group-based gender-transformative intervention on their caregiving responsibilities [29]. Fathers described how through their participation in the intervention, they became more involved in general caregiving and stimulation activities. Fathers who participated in a group-based nurturing care intervention to address child maltreatment and sexual and gender-based violence in peri-urban Uganda also reported increased engagement in general caregiving and stimulation activities [30]. Overall, these findings support the acceptability, feasibility, and positive impact of engaging fathers in caregiving in diverse East African contexts.

These prior studies on nurturing care in various contexts across Eastern and Southern Africa also align with our findings on the role of other family members as caregivers of young children and sources of parenting knowledge. Although all caregivers identified mothers as the primary caregiver of young children because “mothers know best”, they also emphasized that each member of the family had a role to play in caregiving [16, 17, 28] In Malawi, older siblings were responsible for watching and playing with young children when their parents were busy and grandparents – especially grandmothers – mostly engaged in feeding, bathing, and caring for the child [16]. In our sample, aunts were also actively involved in general caregiving activities whereas uncles mostly engaged in stimulation activities. A qualitative study on parenting stress in Tanzania highlighted the important roles that other caregivers had in caring for young children and how this helped to alleviate maternal and paternal parenting stress [31]. Beyond these caregiving roles, these studies highlight the impact that the beliefs and attitudes of other caregivers about parenting may have on how mothers and fathers care for their children.

Indeed, family belief systems about child development and parenting can facilitate or hinder caregivers’ behaviour change in response to an intervention [32]. For example, in the Rwandan and Ugandan interventions, some fathers acknowledged facing resistance from family members who viewed caregiving as a maternal responsibility when they tried to increase their engagement in caregiving activities [29, 30]. In our sample, participants specifically spoke about community resistance to fathers’ engagement in early child health services [12]. Barriers to paternal involvement included maternal-centric attitudes of some health providers who dismissed fathers’ ability to care for their children and cultural norms concerning the gendered division of childcare responsibilities, that contributed to stigmatization of fathers who took their children to health facilities.^12^ Further research is thus needed not only to examine how to leverage the existing role of family support networks in caregiving practices and as sources of parenting knowledge, but also to understand how best to engage other family members in conversations on cultural and social attitudes, beliefs, and norms surrounding caregiving. Additionally, understanding the social, cultural, and economic factors that shape caregiving practices can help identify relevant targets for interventions to promote nurturing care practices.

Our findings highlight the importance of leveraging different sources of parenting knowledge in nurturing care interventions. Caregivers in our sample – especially those with higher levels of education – learned about general caregiving and stimulation activities from facility-based health providers. This was not the case in prior qualitative samples. Caregivers in the Malawi study did not mention health providers as sources of knowledge and caregivers from peri-urban South Africa saw health providers as sources of knowledge about children’s healthcare, but not about stimulation activities [15, 16]. This was also the case for caregivers from other areas of Mozambique, who did not receive any information about stimulation activities from health providers [13, 14]. It is possible that caregivers in our sample reported health providers as sources of knowledge due to the ongoing pilot intervention to improve nurturing care for ECD within the existing health system, whose evaluation this study was embedded in [21].

That the caregivers in our sample identified facility-based health providers as sources of parenting knowledge suggests that training health providers to deliver ECD messaging as part of routine services may help improve parenting knowledge and practices. Given the extensive reach of the health sector to caregivers and children during the crucial period from conception throughout early childhood, integrating key components of successful nurturing care interventions into health systems can facilitate wider uptake of nurturing care practices [33]. It will be important to ensure that these messages are accessible and clearly communicated to all caregivers, regardless of their education level. Furthermore, it is worth noting that although facility-based health providers were sources of parenting knowledge in our study, other health providers or workers in other systems (e.g., education services) should be considered as potential sources of parenting information in other contexts. The Care for Child Development approach provides materials to support the training of health providers to support caregivers in responsive caregiving and early learning activities and improve caregiver-child interactions through responsive play and communication [34]. It has been adapted for use by providers in other sectors (e.g., early childhood care educators) and found to improve parenting practices and ECD outcomes [35]. Caregivers in our sample also described radio programs and posters in health facilities as sources of parenting knowledge, highlighting the potential of audio-visual media as useful strategies for promoting ECD. Understanding whom caregivers turn to for guidance and support about ECD in a given context can help inform the process of determining who can effectively deliver nurturing care interventions [19].

Our findings should be interpreted in the context of certain limitations. Given that this study was embedded in a larger qualitative evaluation of a pilot, we were unable to modify the interview guides and sampling frame in accordance with our research question. The available sample largely consisted of mothers, and we therefore had little data from fathers’ perspectives and no data from other family members. Fathers may also have not felt comfortable sharing their perspectives as most interviewers were female. Additionally, the sample does not include adolescent parents, who are likely to have different caregiving experiences [36]. Future research should include greater representation of fathers and other caregivers of different ages and backgrounds (e.g., different levels of education) to confirm saturation and generalizability of maternal perspectives and gain a more nuanced understanding of the caregiving roles of different family members, including how caregiving practices are shaped by contextual factors. Furthermore, participants’ recent exposure to a pilot intervention that focused on promoting nurturing care practices and ECD in child health services likely had an impact on their knowledge and parenting practices (see Supplementary Material) [12]. Given the novelty of this pilot and its specificity to the Monapo district, our findings may not be generalizable to other similar contexts. Specifically, as these data were collected from caregivers who had recently been exposed to a pilot public health intervention to improve nurturing care for ECD, participants’ responses may be specific to their experiences of the intervention.

## Conclusion

Our findings on how mothers and fathers in rural Mozambique care for their children and from whom they receive guidance and support in providing nurturing care underscore the importance of understanding diverse sociocultural contexts and how they can inform nurturing care practices [4]. This and other studies that describe the lived experiences of caregivers across various sub-Saharan African contexts can help inform the design of nurturing care interventions that align with the social and cultural context of children and their caregivers.

### Supplementary Information

Supplementary Material 1.

Supplementary Material 2.

## Data Availability

The data analyzed during this study are included in this article. However, as our data were collected using qualitative research methods from a small group of participants, there is a risk that study participants may be identifiable. Additional data may be available to access upon reasonable request and for researchers who meet the criteria for access to confidential data. Data requests can be sent to the Managing Director of the Harvard T.H. Chan School of Public Health Office of Human Research Administration: Leslie Howes, MPH, CIP, Managing Director. Email: lhowes@hsph.harvard.edu.

## Abbreviation

ECD: Early childhood development

## References

[1] WHO, UNICEF, Bank W. Nurturing care for early childhood development: a framework for helping children survive and thrive to transform health and human potential. Published online. 2018.

[2] Aboud FE, Yousafzai AK (2015). Global health and development in early childhood. Annu Rev Psychol.

[3] Jeong J, Franchett EE, de Oliveira CVR, Rehmani K, Yousafzai AK (2021). Parenting interventions to promote early child development in the first three years of life: A global systematic review and meta-analysis. PLoS Med.

[4] Draper CE, Barnett LM, Cook CJ (2023). Publishing child development research from around the world: An unfair playing field resulting in most of the world’s child population under-represented in research. Infant Child Dev.

[5] Aboud FE, Yousafzai AK. Very Early Childhood Development. In: Black R, Laxminarayan R, Temmerman M, Walker N, editors. Disease Control Priorities, Third Edition (Volume 2): Reproductive, Maternal, Newborn, and Child Health. Washington D.C.: The World Bank; 2016. 27227205

[6] Eshel N, Daelmans B, de Mello MC, Martines J (2006). Responsive parenting: interventions and outcomes. Bull World Health Organ.

[7] MISAU/Moçambique M da S, INE/Moçambique IN de E, International ICF. Moçambique Inquérito Demográfico e de Saúde 2011. Published online March 1, 2013. https://dhsprogram.com/publications/publication-fr266-dhs-final-reports.cfm. Accessed July 12, 2021.

[8] Instituto Nacional de Estatística (INE) e ICF. Inquérito Demográfico e de Saúde em Moçambique 2022–23. Maputo, Rockville: INE e ICF; 2024.

[9] de Araujo SN, Dade A, Zacarias M de F, Chipembe CS, Maunze XH, Singano CC. Final Report of the Multiple Indicator Cluster Survey, 2008. National Statistics Institute; 2008. https://mics-surveys-prod.s3.amazonaws.com/MICS3/Eastern%20and%20Southern%20Africa/Mozambique/2008/Final/Mozambique%202008%20MICS_English.pdf. Accessed 19 April 2021.

[10] Jeong J, McCoy DC, Yousafzai AK, Salhi C, Fink G (2016). Paternal stimulation and early child development in low-and middle-income countries. Pediatrics.

[11] Cuartas J, Jeong J, Rey-Guerra C, McCoy DC, Yoshikawa H (2020). Maternal, paternal, and other caregivers’ stimulation in low- and- middle-income countries. PLoS One.

[12] Jeong J, Ahun MN, Bliznashka L, Velthausz D, Donco R, Yousafzai AK. Barriers and facilitators to father involvement in early child health services: A qualitative study in rural Mozambique. Soc Sci Med. Published online. 2021. 10.1016/j.socscimed.2021.114363. 10.1016/j.socscimed.2021.114363 34500322

[13] da Costa AB, Carreira F, Naran A. Qualitative study on knowledge, attitudes and practices related to interactions between caregivers and children between 0 and 2 years of age in Mozambique. Maputo: KPMG Auditores e Consultores SA; 2018.

[14] Jones L (2012). Report on an ethnographic study of child rearing in Cabo Delgado Province.

[15] Ayob Z, Christopher C, Naidoo D (2021). Exploring caregivers’ perceptions on their role in promoting early childhood development. Early Child Dev Care..

[16] Gladstone M, Phuka J, Mirdamadi S (2018). The care, stimulation and nutrition of children from 0–2 in Malawi—Perspectives from caregivers; “Who’s holding the baby?”. PLOS ONE..

[17] Hollowell J, Dumbaugh M, Belem M (2019). ‘Grandmother, aren’t you going to sing for us?’ Current childcare practices and caregivers’ perceptions of and receptivity to early childhood development activities in rural Burkina Faso. BMJ Glob Health.

[18] Serpell R, Marfo K (2014). Some long-standing and emerging research lines in Africa. New Dir Child Adolesc Dev.

[19] Bentley ME, Johnson SL, Wasser H (2014). Formative research methods for designing culturally appropriate, integrated child nutrition and development interventions: An overview. Ann N Y Acad Sci.

[20] Creswell JW, Poth CN. Qualitative inquiry and research design: choosing among five approaches. California: Sage publications; 2016.

[21] Jeong J, Bliznashka L, Ahun MN (2021). A pilot to promote early child development within health systems in Mozambique: a qualitative evaluation. Published online.

[22] Bliznashka L, Ahun MN, Velthausz D (2022). Effects of COVID-19 on child health services utilization and delivery in rural Mozambique: a qualitative study.

[23] Tong A, Sainsbury P, Craig J (2007). Consolidated criteria for reporting qualitative research (COREQ): a 32-item checklist for interviews and focus groups. Int J Qual Health Care.

[24] Ministério da Saúde (MISAU), Instituto Nacional de Estatística (INE), ICF Internacional. Inquérito de Indicadores de Imunização, Malária e HIV/SIDA Em Moçambique 2015; 2015.

[25] Picolo M, Barros I, Joyeux M (2019). Rethinking integrated nutrition-health strategies to address micronutrient deficiencies in children under five in Mozambique. Matern Child Nutr.

[26] Bazeley P, Jackson K. Qualitative Data Analysis with NVivo. California: SAGE publications limited; 2013.

[27] Schreier M, Flick U (2014). Qualitative content analysis. The SAGE handbook of qualitative data analysis.

[28] Jeong J, McCann JK, Alsager A (2023). Formative research to inform the future design of a multicomponent fatherhood intervention to improve early child development in Mwanza. Tanzania Soc Sci Med.

[29] Doyle K, Kato-Wallace J, Kazimbaya S, Barker G (2014). Transforming gender roles in domestic and caregiving work: preliminary findings from engaging fathers in maternal, newborn, and child health in Rwanda. Gend Dev.

[30] Siu GE, Wight D, Seeley J (2017). Men’s involvement in a parenting programme to reduce child maltreatment and gender-based violence: formative evaluation in Uganda. Eur J Dev Res.

[31] Ahun MN, Jeong J, Kieffer MP, Mwanyika-Sando M, Yousafzai AK (2021). Maternal and paternal perspectives on parenting stress in rural Tanzania: A qualitative study. SSM - Ment Health.

[32] Tomlinson M, Rahman A, Sanders D, Maselko J, Rotheram-Borus MJ (2014). Leveraging paraprofessionals and family strengths to improve coverage and penetration of nutrition and early child development services. Ann N Y Acad Sci.

[33] Richter LM, Daelmans B, Lombardi J (2017). Investing in the foundation of sustainable development: pathways to scale up for early childhood development. The lancet.

[34] UNICEF, WHO. Care for Child Development: Improving the Care of Young Children. 2012.

[35] Ahun MN, Aboud F, Wamboldt C, Yousafzai AK (2023). Implementation of UNICEF and WHO’s care for child development package: lessons from a global review and key informant interviews. Front. Public Health.

[36] Kaye DK (2008). Negotiating the transition from adolescence to motherhood: coping with prenatal and parenting stress in teenage mothers in Mulago hospital. Uganda BMC Public Health.

