# Supplementary material for: A qualitative study of maternal and paternal parenting knowledge and practices in rural Mozambique
Source: BMC Public Health. 2024 Jul 3;24:1778. doi: 10.1186/s12889-024-19291-2 (PMC11223379; doi:10.1186/s12889-024-19291-2)
Supplement: Supplementary file 2 — Supplementary Material 2. [file 12889_2024_19291_MOESM2_ESM.docx]

**SUPPLEMENTARY MATERIAL**

Summary of pilot intervention to improve nurturing care for early child health and development within existing health system in Monapo district, Mozambique (Jeong et al., 2022)

**Nurturing care for ECD pilot in Monapo District, Mozambique (2018-2020)**

***Objective*** – To reinforce nurturing care interventions within existing health services by:

- Strengthening capacity of subnational health system actors to deliver developmental monitoring, nutritional screening, and counseling for early learning and nutrition
- Increasing family awareness of nurturing care services
- Improving provider and caregiver knowledge, attitudes, and practices regarding nurturing care
- Contributing to improved ECD and nutrition outcomes in the first 3 years of life

***Components and delivery agents***

|  | **Health facility-based** | **Community-based** |
| --- | --- | --- |
| Delivery agents | MCH nurses, well-child and sick-child consultation providers, nutritionist, health facility director, specialist providers | Community health workers, providers supporting HIV-affected children, providers supporting children with disabilities |
| Components | Developmental and nutritional monitoring | Developmental and nutritional monitoring |
|  | Messages and counseling on early learning, responsive care, and infant and young child feeding | Messages and counseling on early learning, responsive care, and infant and young child feeding |
|  | Videos and radio sessions in waiting rooms | Use of radio as part of counseling as well as broader community campaigns |
|  | Posters in waiting rooms and consultation rooms | Referrals for at-risk children to health facilities and community-based rehabilitation for children with confirmed delays or disabilities |
|  | Monitoring of nurturing care indicators as part of routine data collection and health management information systems |  |

***Qualitative impact***

- The pilot intervention was delivered with fidelity to the intended model. There were generally high levels of engagement across respondents and acceptability of integrating nurturing care within routine health services.
- There were multiple benefits of the pilot for the health system (e.g., enhanced supervision, streamlines service delivery), health providers (e.g., increased knowledge and skills pertaining to nurturing care), and families (e.g., increased knowledge and practices about nurturing care, greater father involvement).
- Four areas for improvement in future programming were identified: prioritizing counseling sessions over media-based approaches; systems strengthening to address resource constraints; enhancing content and theoretical focus of ECD; and strengthening referral system between health facility and community-based services.

Abbreviations used: ECD – early child development. MCH – maternal and child health.
